# Shining a Light on Venom-Peptide Receptors: Venom Peptides as Targeted Agents for In Vivo Molecular Imaging †

**DOI:** 10.3390/toxins16070307

**Published:** 2024-07-04

**Authors:** Chun Yuen Chow, Glenn F. King

**Affiliations:** 1 Institute for Molecular Bioscience, The University of Queensland, St. Lucia, QLD 4072, Australia; 2 Australia Research Council Centre of Excellence for Innovations in Peptide and Protein Science, The University of Queensland, St. Lucia, QLD 4072, Australia

**Keywords:** venom peptide, ion channel, molecular imaging, fluorophore, peripheral nerve injury

## Abstract

Molecular imaging has revolutionised the field of biomedical research by providing a non-invasive means to visualise and understand biochemical processes within living organisms. Optical fluorescent imaging in particular allows researchers to gain valuable insights into the dynamic behaviour of a target of interest in real time. Ion channels play a fundamental role in cellular signalling, and they are implicated in diverse pathological conditions, making them an attractive target in the field of molecular imaging. Many venom peptides exhibit exquisite selectivity and potency towards ion channels, rendering them ideal agents for molecular imaging applications. In this review, we illustrate the use of fluorescently-labelled venom peptides for disease diagnostics and intraoperative imaging of brain tumours and peripheral nerves. Finally, we address challenges for the development and clinical translation of venom peptides as nerve-targeted imaging agents.

## 1. Introduction

Living organisms orchestrate an incredibly complex medley of biochemical and cellular events, which makes it challenging to unravel these processes. In the past, our progress towards this goal had been hampered by the inability to study these processes without perturbing their native environment. Molecular imaging has revolutionised the field by allowing visualisation and characterisation of biological phenomena at the cellular level in intact living subjects in real time [1,2]. This provides valuable anatomical information and enables the detection of abnormalities, which can potentially facilitate earlier diagnosis and better treatment of disease.

Ion channels constitute a diverse class of membrane proteins that control the passage of ions in and out of cells. Ion transport is a key component in many biological processes such as nerve and muscle excitability, sensory and cognitive function, and signal transduction [3,4]. Inherited or acquired dysfunctions of ion channels are associated with a wide range of diseases, collectively known as channelopathies, across cardiovascular, neuronal, neuromuscular, metabolic, and respiratory systems. Approximately 20% of biologic and small-molecule drugs target ion channels, making them the most common class of biologic drug targets and the second most common class of small-molecule drug targets after G-protein coupled receptors [5,6]. There are more than 400 ion channel genes in the human genome, but the repertoire of functional ion channels is much larger due to alternative splicing. Despite this remarkable molecular diversity, only a few dozen ion channels are targeted by currently available drugs [7]. This highlights an underexploited space for ion channels as therapeutic or theranostic targets.

Patch-clamp electrophysiology is considered the gold standard for the study of ion channels as it allows control of the intra- and extracellular environment and the membrane potential. However, it is difficult to implement in regions with limited accessibility to patch pipettes, it requires specialised equipment and sophisticated skills, and it does not provide information about the in vivo distribution of ion channels. Changes in ion channel expression and distribution could underlie several neurological disorders such as nerve injuries. With an appropriate imaging agent, molecular imaging offers a non-invasive method to study the expression and localisation of ion channels in living cells, tissues, experimental animals, or human patients in real time.

### 1.1. A Quick Look at Molecular Imaging Agents

**Figure 1:**
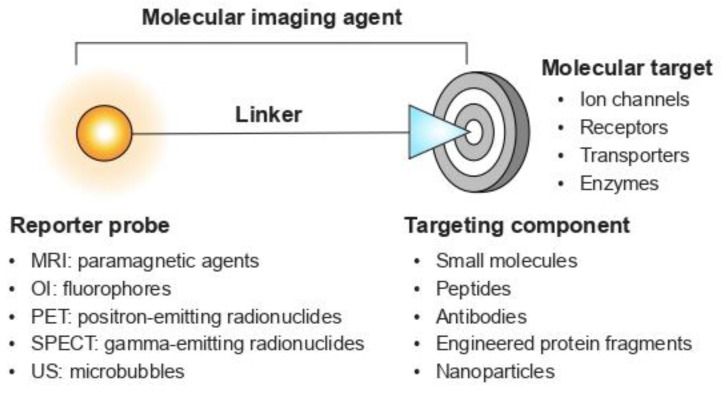
Schematic representation of a molecular imaging agent. An imaging agent typically consists of a reporter probe and a targeting component. The optimal reporter probe is determined by the imaging modality employed. Different imaging modalities exist, including magnetic resonance imaging (MRI), optical fluorescence imaging (OI), positron emission tomography (PET), single-photon emission computed tomography (SPECT), and ultrasound (US). Each of these imaging modalities has its own strengths and limitations in terms of sensitivity, spatial resolution, tissue-penetration depth, and cost [9].

Molecular imaging generally requires an imaging agent as the source of image contrast. An imaging agent should have the following properties to accurately visualise the desired target (e.g., an ion channel or neuronal receptor): high selectivity for the target of interest, suitable pharmacokinetic profile, good in vivo stability, favourable safety profile, time- and cost-effective production, and multiplexing capabilities [8]. Molecular imaging agents typically comprise a reporter probe and a targeting component (Figure 1). In general, a reporter probe is chemically linked to a targeting component that interacts with the target of interest [8]. Common classes of targeting components include small molecules, peptides, and monoclonal antibodies (mAbs).

Small molecules (typically <500 Da) offer good pharmacokinetic properties such as rapid accumulation in the target tissues and fast clearance rate. However, their small size also means that they often have poor specificity and are subject to limitations on the type of reporter probe that can be attached [10]. Attachment of a large fluorescent dye can alter the binding properties and pharmacokinetics of a small molecule. In contrast, mAbs (~150 kDa) can accommodate large fluorophores without altering their specificity and affinity towards their molecular target or their pharmacokinetics. However, mAbs have biological half-lives of days to weeks, which are far from optimal for imaging purposes. Prolonged exposure of labelled mAbs in non-target tissues can lead to low signal-to-background ratio and poor imaging resolution [11]. Moreover, it has proven difficult to identify antibodies for many ion channels due to the complexity of their membrane-embedded structures [12,13]. For example, voltage-gated ion channels typically have very few extracellular regions that contain only a limited number of potential epitopes, making them less accessible for antibody binding. Peptides offer the benefit of small molecules, such as rapid clearance kinetics and high tissue-penetration depths, while retaining target specificity and the low immunogenicity characteristic of mAbs [14,15]. In addition, peptides can be synthesised using solid-phase synthesis, and consequently, they offer superior flexibility for introduction of chemical modifications to improve their biological activity, solubility, and stability.

### 1.2. Animal Venoms: A Rich Source of Imaging Agents

It has been estimated that ~15% of extant animal species are venomous [16], ranging from minute flies and pseudoscorpions to large snakes and venomous mammals such as the platypus and solenodons. This incredible diversity of venomous animals has resulted in a vast number of venom components with extremely diverse pharmacology [17]. The primary components of most arthropod venoms are disulfide-constrained peptides. These peptides primarily target ion channels and neuroreceptors. Disulfide-constrained venom peptides are attractive leads for drug discovery owing to their high selectivity, potency, and stability [16,18]. With advances in high-throughput screening techniques and comprehensive transcriptomic and proteomic analyses, venom peptides have expanded our understanding of the physiological roles of ion channels in their native cellular environment. As of today, there are six venom-derived active ingredients approved for clinical use by the U.S. FDA, with many more undergoing clinical trials and preclinical development [17]. One example is ziconotide, an analgesic used for the treatment of intractable chronic pain. This 25-residue peptide from venom of the cone snail *Conus magus* is an antagonist of the N-type voltage-gated calcium channel (Ca_V_2.2) [19].

Many naturally occurring venom peptides have high affinity and specificity for their cognate target, rendering them ideal targeting components for imaging applications. Radiolabelling and fluorescent labelling are the two primary methods of adapting venom peptides for imaging applications. The former was widely used in the 1980s to 1990s [20,21,22] prior to the development of fluorescent techniques. For example, radiolabelled charybdotoxin and apamin, peptides from venoms of the scorpion *Leiurus quinquestriatus hebraeus* and the honeybee *Apis mellifera*, respectively, were utilised to map the distribution of large-conductance and small-conductance calcium-activated potassium (K_Ca_) channel in rat brain [23]. Alternatively, venom peptides can be conjugated with a fluorophore in a site-specific manner using a wide range of chemical strategies such as amine labelling and click-chemistry-based approaches [24,25]. Fluorescently labelled venom peptides have played an important role in defining the functional characteristics of ion channels in living cells and tissues (Table 1). For example, fluorescent derivatives of HgTx1, a peptide isolated from venom of the scorpion *Centruroides limbatus*, were used to examine the localisation of K_V_1 family channels in rat cerebellum using fluorescence microscopy [26].

### 1.3. Near-Infrared Fluorescent Imaging

**Figure 2:**
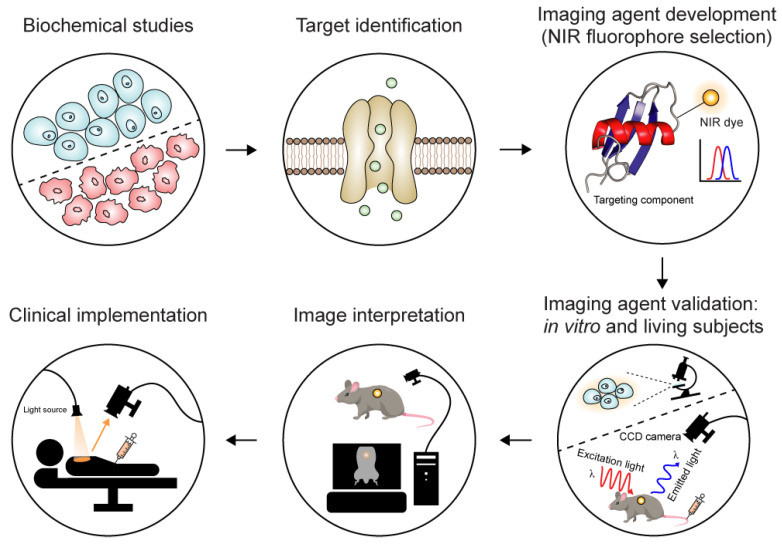
Schematic showing the key stages involved in NIR fluorescence imaging. In brief, the first step is to study the biochemical process of interest and assess the possibility of visualising this process via molecular imaging techniques. The second step is to determine the molecular target involved in the process of interest, followed by selection of an appropriate molecular imaging agent that has a NIR fluorophore attached to a targeting component. Subsequent in vitro and in vivo studies are required to evaluate the specificity and affinity of the imaging agent. During imaging, the subject is illuminated with excitation light of an appropriate wavelength, and the subsequent light emission is detected by a charge-coupled device (CCD) camera. The signal is then converted into an image that indicates the location of the emitted light (i.e., from the imaging agent) within the subject. After validation, the imaging agent enters early-stage clinical trials. The imaging agent might lead to new insights during clinical trials and cause the cycle to begin again.

Alongside the development of fluorescent labelling approaches and imaging instrumentation, several macroscopic optical imaging methods provide whole-body imaging of living small animals. Near-infrared (NIR) fluorescent imaging has become popular in preclinical and clinical settings because of its excellent sensitivity, high spatial resolution, ease of use, low cost, and multiplexing capabilities (Figure 2) [54,55]. This technique relies on fluorophores that emit light when excited by a specific wavelength of light within the NIR spectral window (~650–1700 nm) [56]. This approach takes advantage of the fact that absorption of light by endogenous biomolecules such as water, cytochromes, and haemoglobin is lower in the NIR window. Thus, imaging performance can be enhanced by reducing the extent of tissue scattering and autofluorescence (i.e., fluorescent signal from tissues where the imaging agent is not present [57]).

There has been an explosion over the past decade of proof-of-principle studies in the fluorescent imaging of biomarkers, blood vessels, and nerves. There is also growing interest in the use of fluorescence-guided surgery, an intraoperative imaging method that provides non-invasive assessment of normal and diseased tissues [58]. It can be used to define tumour location [59,60] and ensure the preservation of nerve structures during surgical procedures [61]. In this review, we highlight a selection of in vivo imaging applications using venom-derived peptides and discuss the promise and challenges associated with clinical translation of venom peptides as imaging agents.

## 2. Chlorotoxin: An Intraoperative Agent for Imaging Brain Tumours

Gliomas are the primary tumours that originate from glial cells. They are known for their fast proliferation rate and tendency to invade normal brain tissues [62,63]. Current therapy involves surgical resection, followed by radiation with adjuvant chemotherapy [64]. The success of resection has a significant impact on the prognosis of brain cancers, as more than 80% of malignant tumours recur due to unresected residual tumours [65]. One of the challenges of surgical removal is to identify and visualise the brain tumour tissues from normal cortex. Accurate margin delineation is crucial, particularly in delicate regions, as broader resection in such areas leads to patient morbidity and loss of functionality. Balancing aggressive resection against the necessity to preserve surrounding healthy tissues and minimise functional impairment is the goal of brain tumour surgery. It would, therefore, be beneficial to develop tumour-targeted agents that distinguish between tumours and normal brain tissue. This would potentially preclude the need for invasive procedures such as biopsies.

### 2.1. Chlorotoxin: From Primary to Tertiary Structure

**Figure 3:**
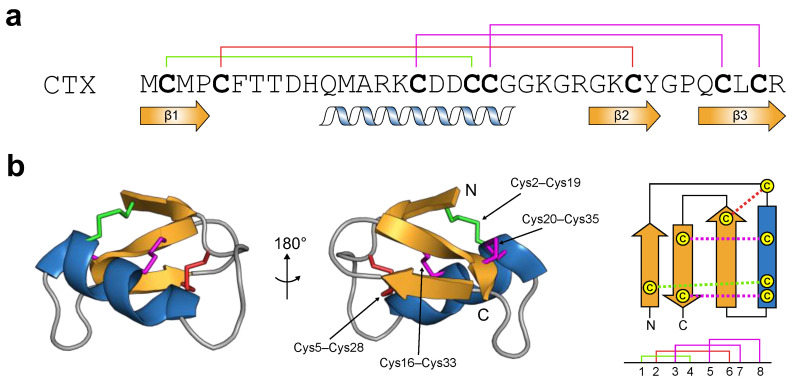
Structure of chlorotoxin (CTX). (**a**) Primary structure of CTX. Cysteine residues characteristic of CSαβ peptides are shown in bold. The disulfide connectivities are colour-coded and illustrated above the sequence. The secondary structural elements of CTX are shown below the sequence. (**b**) Ribbon representation of the solution structure of CTX (PDB: 1CHL) with the corresponding secondary structure topology shown at right. The N- and C-termini are labelled. The disulfide connectivities are coloured according to (**a**).

Chlorotoxin (CTX) from venom of the Israeli scorpion *Leiurus quinquestraitus* is one of the most well-studied venom peptides. It has emerged as an imaging agent due to its preferential binding to glioma cells in the brain and other tumours of neuroectodermal origin [66]. CTX has 36 amino acid residues that are cross-linked by four disulfide bridges (Figure 3a). Its tertiary structure conforms to the cystine-stabilised α-helix/β-sheet (Csαβ) motif [67]. This scaffold is characterised by an α-helix anchored to a double-stranded antiparallel β-sheet via two disulfide bonds (highlighted in magenta; Figure 3b). The majority of Csαβ peptides contain a third disulfide bond that connects the N-terminal region of the α-helix to the second β-strand (highlighted in red for CTX, Figure 3b) [68]. Comparison of the structure of CTX with other Csαβ peptides shows that it has a unique Csαβ fold, making it the canonical member of the ‘chlorotoxin-like peptide’ family. This family is characterised by a fourth disulfide bridge linking the N-terminal region (i.e., in the first β-strand) and the last segment of the α-helix (highlighted in green; Figure 3b).

### 2.2. Chlorotoxin: A Biological Chameleon

CTX was first identified as a blocker of small-conductance chloride ion channel currents in rat epithelia and embryonic rat brain [69,70]. These findings were subsequently confirmed by patch-clamp electrophysiology studies, where CTX inhibited chloride ion channels expressed in cultured glioma cell lines and patient biopsies [71,72,73]. In 1998, Soroceanu and co-workers made a major breakthrough in understanding the tumour-binding properties of CTX. Intracranial injection of radiolabelled CTX led to accumulation in gliomas but not surrounding normal tissues in xenograft mouse models (i.e., mice harbouring human glioma cells) [74]. Later, a recombinant polyhistidine-tagged version of CTX was shown to inhibit matrix metalloproteinase-2 [75], which is upregulated in gliomas but not expressed in normal brain tissues [76]. Annexin A2, a calcium-dependent phospholipid binding protein present on the extracellular side of the plasma membrane of tumours and vascular endothelial cells, has also been proposed as a target for CTX [77]. siRNA knockdown of annexin A2 in pancreatic tumour cells abolished the binding of CTX to the cell surface. While studying the molecular targets for CTX has not been straightforward, the above targets are involved in malignant cell migration and invasion [78]. These findings suggest that inhibiting neural targets that are expressed by gliomas could provide a potential therapeutic strategy to restrict glioma invasion.

### 2.3. Chlorotoxin: Imaging Applications

A variety of CTX-conjugated analogues have been evaluated in preclinical and clinical trials [79,80]. One such molecule is ^131^I-TM-601, an ^131^I-labelled synthetic version of CTX, which has been utilised both as an imaging tool to assess the extent of glioma invasion and as a radiotherapy agent to control tumour progression [81,82]. In a Phase I clinical trial on 18 adult patients with recurrent high-grade gliomas, a single dose of ^131^I-TM-601 was delivered directly into the patient’s brain via an intracavitary reservoir after the surgical resection of gliomas, and it was shown to be well tolerated with no significant toxicity [82]. ^131^I-TM-601 was bound to the gliomas during the five-day monitoring period with no appreciable binding elsewhere in the body, while the remaining unbound peptide was eliminated from the body within 24 to 48 h after administration (clinical trial identifier number: NCT00114309). Unfortunately, this study and a subsequent Phase II trial failed to demonstrate patient survival advantage to justify further trials [81].

Since the discovery of CTX, tremendous efforts have been made to develop CTX-based imaging agents using NIR fluorophores in order to enable precise tumour identification and localisation in real time. CTX was developed into a fluorescent imaging agent by the Olson group through conjugation with a Cy5.5 NIR dye (CTX-Cy5.5) [43]. In a transgenic mouse model of medulloblastoma, CTX-Cy5.5 was shown to selectively bind to gliomas one day after tail vein injection, indicating its ability to permeate the blood–brain barrier (BBB) [43]. CTX-Cy5.5 also delineated tumour margins in mouse models of medulloblastoma, sarcoma, lung metastases, prostate cancer, and intestinal cancer. The unbound CTX-Cy5.5 was rapidly distributed in the mouse body, particularly in the kidney and liver [43]. CTX contains three Lys residues at positions 15, 23, and 27 that serve as reactive sites for the conjugation of Cy5.5 via amine-NHS (*N*-hydroxysuccinimide) ester coupling, resulting in a mixture of mono-, di-, and tri-labelled peptides. To simplify production of the mono-labelled form, CTX was modified by substituting Lys15 and Lys23 with Ala or Arg [83]. In this way, only Lys27 was labelled with Cy5.5 dye, and the product retained the tumour-binding activity and in vivo stability of native CTX, with ~30% of the peptide degraded over 24 h in human serum.

CTX has also been conjugated to cyanine-based fluorescent dyes, including IR800 and indocyanine green (CTX-IR800 and BLZ-100, respectively). IR800 has been used in early-phase clinical trials [57], whereas indocyanine green (ICG) is an FDA-approved fluorophore for several oncological applications, including tumour or sentinel lymph node localisation [84], coronary angiography [85], and metastasectomy [86]. Both CTX-IR800 and BLZ-100 were shown to localise to human glioblastoma cells implanted in mice [41,42], recapitulating the activity of CTX-Cy5.5. BLZ-100 has undergone extensive preclinical studies in small and large animal models harbouring tumours [87,88]. Recent evaluation of BLZ-100 (tozuleristide) in a Phase I clinical trial indicated that it was well-tolerated with no toxicity at doses up to 30 mg (NCT02234297 and NCT02462629) [89]. The fluorescent signals were detected in both low- and high-grade tumours [89], breast carcinomas [90], and skin tumours [91]. A Phase II/III trial evaluating BLZ-100 for fluorescence-guided imaging of paediatric brain tumours (NCT03579602) was completed in June 2022, but the results have not been published.

Overall, CTX-based imaging agents have proven effective for tumour diagnosis and improving tumour resection, which are cornerstones of cancer therapy. However, there is still much to be understood about the mechanism by which CTX breaches the BBB and binds to brain tumours. Several minimised versions of CTX have been designed and evaluated, with the aim of reducing its structural complexity while increasing its BBB shuttle activity [92]. These gaps in knowledge present opportunities for the rational design of peptide variants and optimisation based on the structural scaffold of CTX. The ongoing development of CTX-based imaging agents holds significant promise for improving outcomes for cancer patients.

## 3. Peripheral Nerve Injury

**Figure 4:**
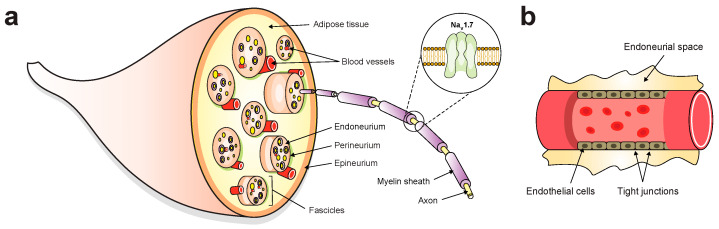
Schematic diagram of peripheral nerve anatomy. (**a**) Myelinated or unmyelinated axons are the basic unit of a peripheral nerve. Neuron axons are supported by three connective tissue sheaths. The innermost sheath is the endoneurium, whereas the outer border is the perineurium. The epineurium is the outermost sheath that envelops the fascicles and blood vessels within adipose tissue. Na_V_1.7 is highly expressed in the peripheral neurons and is mostly restricted to the peripheral nervous system for the transmission of nociceptive signals. For the sake of clarity, the insert shows only Na_V_1.7 channels found at nodes of Ranvier in myelinated axons. (**b**) Structure of endoneurial blood vessels. The blood–nerve barriers are composed of a physical barrier at endoneurial vessels within the fascicle and the investing perineurium. These two barriers act as protective interfaces to allow the entry of nutrients and prevent toxic substances from entering the endoneurial space.

The nervous system coordinates the functions of tissues and organs by processing information from the surrounding environment and governing the responses of organisms to stimuli or stresses. The peripheral nervous system (PNS) is a highly heterogenous entity that provides communication between the brain and the body. Each peripheral nerve has several main components: an outer epineural layer enveloping multiple fascicles, a perineural layer consisting bundles of individual axons, and an inner endoneurial layer covering individual axons (Figure 4a) [93]. Peripheral nerve fibres, particularly at axonal sites distant from the neuronal cell bodies, are filigree structures that are susceptible to damage from compression, trauma, or medical disorders [94,95]. As a result of damage to motor and sensory nerves, the affected individuals experience various clinical manifestations, including allodynia, chronic neuropathy, and prolonged loss of muscle movement and/or sensations [96].

During oncological surgery or after traumatic injury, surgeons often face difficulties in precisely locating and preserving vital nerve structures due to tissue distortion and poor visibility. This can lead to unintended nerve injuries that impose significant physical, psychological, and economic burdens on individuals and their families. While most peripheral nerve injuries are of traumatic origin, approximately 25% of patients attribute their neuropathic pain to surgical intervention [97,98,99]. For example, a common postoperative complication after thyroid surgery is injuries to the recurrent laryngeal nerves, leading to changes or loss of voice or respiratory complications [100]. A common problem with prostatectomy is damage to nerves of the prostatic plexus, which can lead to urinary incontinence or erectile dysfunction [101].

### 3.1. Intraoperative Preservation of Peripheral Nerve Tissues

Intraoperative nerve visualisation is an unmet clinical need. The existing intraoperative tools, such as loupes and light microscopes, are only effective for nerves close to the tissue surface and they are applicable only to certain types of interventions [102]. Electromyography (EMG) is an alternative approach to identify motor nerves before direct surgical exposure, in which stimulating electrodes are placed in the muscle innervated by the target nerve, and muscle activity is monitored [103]. However, EMG does not offer direct visual guidance for surgeons, and it is ineffective if the axonal or neuromuscular transmission distal to the recording sites is blocked. Advanced magnetic resonance imaging (MRI) and ultrasound have also been used to identify large nerve bundles in an intraoperative setting [104,105]. However, these imaging modalities could interrupt the normal surgical workflow and lengthen anaesthesia times, and their use is limited by several factors, including equipment availability and cost. In contrast, nerve-imaging agents have the potential to provide a visual clue for surgeons to delineate peripheral nerves in real time during surgery, thereby reducing surgical morbidity.

An ideal nerve-targeted agent should possess the following properties: high specificity for peripheral nerves, impermeability to the BBB, low systemic toxicity, minimal accumulation in non-target tissues, and fast clearance rate. Fluorescein and ICG are the two FDA-approved fluorophores for indirectly visualising nerves by highlighting the adjacent small blood vessels known as vasa nervorum (Figure 4b). Recently, fluorescein has been used to visualise abnormal cystic peroneal nerves in ganglion cyst excision [106] and to delineate the boundary between tumour tissues and surrounding nerves in neurofibromas and schwannomas [107]. Several clinical trials demonstrated that ICG could be used to identify facial nerves during mastoidectomy [108], neurovascular bundles during radical prostatectomy [109], and pelvic nerves during hysterectomy [110]. These neurovascular dyes remain confined to the vascular space due to the lack of nerve-targeting properties. One of the drawbacks of such an approach is that visualisation is inadequate when the nerves are buried by surrounding tissues, particularly in highly vascular anatomic regions [111], thus restricting widespread use of these dyes in a surgical setting.

To date, three binding sites within nerve fibres have been identified for nerve-imaging agents, including the axon, myelin sheath, and endoneurium [112]. Axon-targeted agents, including neurotropic viral and nonviral tracers, rely on axonal transport mechanisms and require local administration due to their inability to cross the blood–nerve barrier (BNB; Figure 4b) [113]. These tracers also require an extended incubation period due to their slow axonal transport, which presents practical challenges for intraoperative use. Several small molecules and oligopeptides have been designed and reported to bind to the myelin sheath and endoneurium, in which the myelin basic protein and proteoglycans are the potential molecular targets, respectively [114,115,116,117]. An example is NP41, a 12-residue peptide, which is localised to the endoneurium of the nerves in mice after systemic injection, as well as resected human recurrent laryngeal nerves [118]. In addition, NP41 has been used to highlight facial nerves in mice [119] and autonomic nerves within the rat prostate [120]. Importantly, NP41 cannot bypass the BBB, therefore avoiding potential issues of central toxicity and accumulation [118].

### 3.2. Na_V_1.7: Contribution of Peripheral Nerves

Voltage-gated sodium (Na_V_) channels play a fundamental role in normal neurological function, especially in the initiation and propagation of action potentials in neurons, muscles, and other fast signalling tissues [121]. Nine Na_V_ channel subtypes have been identified in mammals, denoted Na_V_1.1–1.9. The main subtypes expressed in normal adult peripheral sensory neurons are Na_V_1.1, Na_V_1.6, Na_V_1.7, Na_V_1.8, and Na_V_1.9. The latter three are crucial in generating and transmitting nociceptive signals. Among these three isoforms, Na_V_1.7 can be found in small diameter dorsal root ganglion neurons [122,123]. Neurons expressing Na_V_1.7 are capable of amplifying slowly developing subthreshold depolarising inputs, and hence, this channel subtype directly modulates the action potential threshold in nociceptors [124,125].

The Na_V_ channel α-subunit is a single polypeptide chain that organises into four non-identical domains (DI to IV), each containing six transmembrane segments (S1–S6). The S1 to S4 segments in each domain constitute a voltage-sensing domain, wherein S4 contains positively-charged residues essential for voltage sensing in response to changes in membrane potential. The S5 and S6 segments from each domain come together in a circular arrangement to form the channel pore and the selectivity filter that determines sodium ion selectivity. A number of high-resolution cryoEM structures of human (h) Na_V_ channels have been reported [126,127,128,129,130,131,132,133]. Taken together, structures of the resting-state, open-state, and multiple ligand-bound states have provided fundamental insights into the molecular mechanisms of voltage-sensing, fast inactivation, sodium permeation, and modulation of channel activity by diverse ligands.

The fundamental role of Na_V_1.7 in pain processing was revealed by seminal human genetic studies. First, gain-of-function mutations in the gene *SCN9A* that encodes the α-subunit of Na_V_1.7 were shown to underlie inherited pain disorders such as erythromelalgia and paroxysmal extreme pain disorder [134,135]; these syndromes are characterised by burning pain and redness in the extremities. Then, in 2006, it was shown that loss-of-function mutations in *SCN9A* lead to a congenital inability to sense pain [136]. The loss of Na_V_1.7 function does not cause intellectual disability or any sensory impairments aside from anosmia. Thus, Na_V_1.7 has received a tremendous amount of attention as a potential analgesic drug target [137]. Unfortunately, many Na_V_1.7-targeted drug candidates showed efficacy in preclinical animal models but failed to translate to the clinic. There are many reasons that possibly contribute to this failure, including the lack of selectivity of some compounds for Na_V_1.7 over other Na_V_ channel subtypes, the absence of pharmacology-altering β-subunits in pharmacological assays [138], and the use of rodent pain models with limited relevance to human chronic pain to assess analgesic efficacy [139]. However, regardless of its therapeutic relevance, Na_V_1.7 is an ideal target for imaging purposes as it is selectively expressed on most peripheral neurons.

### 3.3. Spider-Venom Peptides as Nerve-Targeted Agents

Spider-venom peptides have proven to be excellent pharmacological tools for structure-function analysis of Na_V_ channels and dissection of their (patho)physiological roles. There are twelve families of spider-venom peptides that modulate Na_V_ channels (known as NaSpTx), based on their primary structures and disulfide-bond connectivities [140]. Most spider-venom peptides are allosteric modulators known as gating modifiers that bind to one or more of the voltage-sensing domains in order to alter channel activation or inactivation. An example is the 35-residue peptide HwTx-IV, a member of NaSpTx family 1, which acts as a channel inhibitor by interacting with the voltage-sensing domain in DII to stabilise a resting conformation of Na_V_1.7 [141]. The majority of spider-venom peptides range in size from 3.0–4.5 kDa. They typically adopt a stable inhibitor cystine knot fold, wherein one of the disulfide bonds bisects a loop formed by the other two disulfides and the intervening sections of the peptide backbone [142]. This so-called knottin framework greatly enhances resistance to chemical, thermal, and proteolytic degradation. Below we discuss two spider-venom peptides that target Na_V_1.7 and that have been used for peripheral nerve visualisation.

#### 3.3.1. μ-theraphotoxin-Hs1a

**Figure 5:**
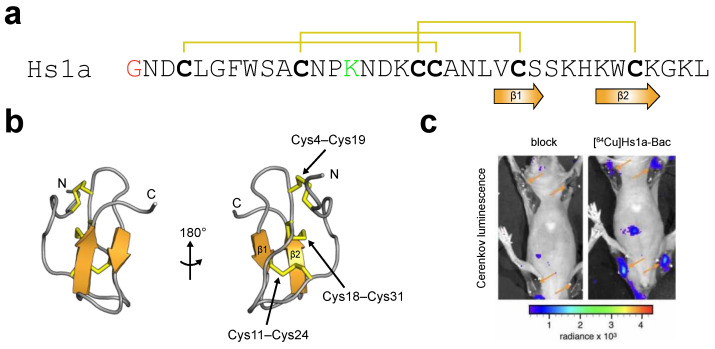
Use of Hs1a for intraoperative nerve imaging. (**a**) Primary structure of Hs1a. Cysteine residues characteristic of the inhibitor cystine knot motif are shown in bold. A non-native N-terminal Gly residue (highlighted in red) was added to the Hs1a coding sequence to optimise its cleavage from the fusion protein used to facilitate recombinant expression of the peptide. For imaging studies, a Cy7.5 dye or bacteriochlorin was attached to Lys14 (highlighted in green). (**b**) Ribbon representation of Hs1a (PDB: 2MT7). The N- and C-termini are labelled. The antiparallel β strands are coloured orange, whereas the three disulfide bonds are shown in yellow. (**c**) Cerenkov luminescence images of mice injected with a ‘blocking’ formulation (unlabelled Hs1a and ^64^Cu-labelled Hs1a-Bac; left) or ^64^Cu-labelled Hs1a-Bac (right) showing the non-invasive visualisation of brachial plexus and sciatic nerves, as indicated by the orange arrows. [Figures from [50] Copyright 2023, American Chemical Society].

A recombinant version of μ-theraphotoxin-Hs1a (henceforth Hs1a; Figure 5a,b), which is found in venom of the Chinese tarantula *Cyriopagopus schmidti*, was conjugated to NIR dye Cy7.5 to monitor the distribution of Na_V_1.7 in mouse sciatic nerves ex vivo, as confirmed by immunohistochemistry [49]. A recent paper by Hernández-Gil and co-workers reported the labelling of Hs1a with a bacteriochlorin fluorophore (Hs1a-Bac) [50]. Bacteriochlorin is a member of the porphyrin family; it has two diagonally opposed pyrrole rings that can chelate radionuclides for use with different imaging modalities. Intravenous (IV) injection of Hs1a-Bac leads to high uptake in the peripheral nerves of mice. Biodistribution studies revealed that the resulting probe accumulated in the spleen, liver, and kidney but not other organs such as the muscles, heart, and brain [50].

Radiolabelling Hs1a-Bac with ^64^Cu provides a strategy for rapid assessment of nerve damage and intraoperative surgical intervention by positron emission tomography/computed tomography (PET-CT) and Cerenkov luminescence (CL) imaging, respectively. The inherent Cerenkov radiation produced by ^64^Cu allows intraoperative nerve identification using optical imaging. This multimodal approach maximises comparability between PET-CT and optical imaging, thus minimising the number of injections. IV injection of ^64^Cu-labelled Hs1a-Bac produced adequate fluorescent signals in the brachial plexus and sciatic nerves in whole-body CL images (Figure 5c) [50]. Clinically, brachial plexus injuries commonly occur during coronary bypass procedures and shoulder debridement, whereas sciatic nerve injuries pose a risk to patients undergoing hip or knee replacement surgery [112]. Together, this study demonstrated the feasibility of using a Na_V_1.7-targeted venom peptide as a NIR multimodal imaging agent and validated the neural specificity and fluorescent imaging properties of ^64^Cu-labelled Hs1a-Bac. In addition to providing a starting point for nerve visualisation, Hs1a offers valuable insights into the complex anatomy and function of the PNS. Despite these promising data, little is known about the overall safety of ^64^Cu-labelled Hs1a-Bac. Future directions should include evaluation of its clearance rate (pharmacokinetics), in vivo metabolism, and whether it exerts any toxic effects in mice over extended periods of time.

Electrophysiological studies revealed that Hs1a inhibits multiple hNa_V_ channel subtypes at low nanomolar potency, including Na_V_1.1, Na_V_1.2, Na_V_1.3, Na_V_1.6, and Na_V_1.7 [49]. Both Na_V_1.4 and Na_V_1.5 are critical off-targets for a peripherally administered nerve-imaging agent, as inhibition of these subtypes would alter the contractility of skeletal and cardiac muscles with potentially fatal consequences. Inhibition of Na_V_1.2 is not of major concern as it is exclusively expressed in the brain [143] and Hs1a should not penetrate the BBB after IV administration, as demonstrated by previous biodistribution studies [50]. Na_V_1.3 is prominent in embryonic neurons but its expression is significantly attenuated during postnatal development and is, therefore, not present in healthy adults [144]. Conversely, both Na_V_1.1 and Na_V_1.6 are broadly distributed throughout the nervous system. Na_V_1.1 is expressed in peripheral Aδ sensory neurons, which are involved in the transduction of mechanical pain [145]. Na_V_1.6 is primarily localised to nodes of Ranvier in myelinated axons, along the full axon in unmyelinated neurons, and in dendrites of projection neurons [146,147]. Consequently, inhibiting Na_V_1.6 poses the risk of blocking nerve transduction, resulting in paralysis. Thus, a crucial step in the development pathway for Hs1a as a nerve-targeted agent would involve the rational engineering of more selective analogues.

#### 3.3.2. μ-theraphotoxin-Tsp1a

**Figure 6:**
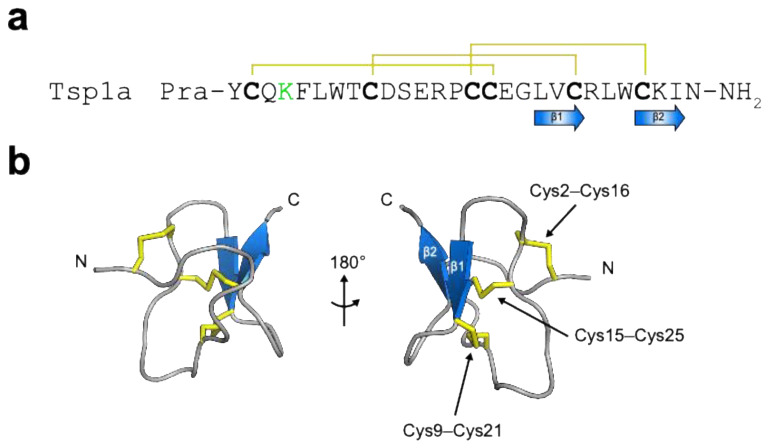
Tsp1a, a candidate for intraoperative nerve imaging. (**a**) Primary structure of Tsp1a. Cysteine residues characteristic of the inhibitor cystine knot motif are shown in bold. Propargylglycine (Pra) was added to the N-terminus of Tsp1a for copper-catalysed click chemistry, whereas the NHS fluorophore was attached to Lys4 (highlighted in green) via amine-NHS ester coupling. (**b**) Ribbon representation of Tsp1a (PDB: 7A64). The N- and C-termini are labelled. The antiparallel β strands are coloured blue, whereas the three disulfide bonds are shown in yellow.

While conducting a screen to identify venom components that inhibit hNa_V_1.1 channel, Jiang and co-workers discovered a 28-residue peptide known as μ-theraphotoxin-Tsp1a (henceforth Tsp1a; Figure 6a,b) from the venom of a Peruvian tarantula (*Thrixopelma* spec.) that potently inhibits Na_V_1.7 [148]. Electrophysiological studies on a panel of hNa_V_ channel subtypes revealed that Tsp1a is one of the most potent and selective peptide antagonists of Na_V_1.7 reported to date (IC_50_ 10 nM), with 24-fold selectivity over Na_V_1.2, 45-fold selectivity over Na_V_1.1, and 100-fold selectivity over Na_V_1.3−Na_V_1.6. Sequence alignments revealed that Tsp1a belongs to NaSpTx family 3. Tsp1a decreases Na_V_1.7 peak currents and induces a hyperpolarising shift in the voltage dependence of steady-state inactivation without altering the conductance–voltage relationship [148], presumably by interacting with neurotoxin binding site 3 (i.e., DIV S3–S4) and stabilising the voltage-sensing S4 helix in a non-conducting inactivated state.

The exquisite selectivity of Tsp1a for Na_V_1.7 raises the question of whether it could be developed as a nerve-targeted agent for intraoperative imaging. A synthetic version of Tsp1a has been conjugated to a BODIPY fluorophore and a chlorin moiety (Tsp1a-BODIPY and Tsp1a-ChL, respectively) [52,53]. While the toxicity of Tsp1a-BODIPY was not determined, no changes in behaviour or apparent toxicity (oxygen level and heart rate) were observed following IV injection of Tsp1a-ChL in mice, indicating a good safety profile [53]. Both Tsp1a-BODIPY and Tsp1a-ChL produced strong fluorescent signals in sciatic nerves after IV injection in mice. Traditionally, in vitro fluorescent imaging relies on dyes with the emission within the visible light range, which can be detected by the human eye. In contrast, for in vivo fluorescent imaging, wavelengths below ~600 nm (i.e., orange to red) encounter limited tissue penetration and high autofluorescence, owing to strong tissue scattering and strong absorbance of haemoproteins (e.g., haemoglobin and cytochromes) [58]. Thus, the BODIPY fluorophore, which emits in the visible range (~510 nm), is not suitable for whole-body imaging, particularly in large mammals such as humans.

Recently, Gonzales and co-workers constructed a library of fluorescently labelled Ts1pa derivatives and evaluated their potential for in vivo imaging [149]. Five NIR fluorophores (IR800, Janelia669, BODIPY665, DY684, and Cy7.5) were selected and conjugated to Tsp1a either via NHS ester coupling to the Lys4 residue or by using copper-catalysed click chemistry to attach the fluorophore to an unnatural propargylglycine amino acid added to the N-terminus (denoted Tsp1a-Pra). Among these analogues, Tsp1a-Pra-IR800 not only retained the inhibitory effect on Na_V_1.7 but also produced the highest fluorescent signal in the sciatic nerves of mice following IV injection. Subsequent biodistribution studies revealed that Tsp1a-Pra-IR800 localises primarily to nerves, with weak fluorescence detected in the liver and other organs. Extensive toxicity experiments were also performed for two weeks after injection, and no toxicity was observed.

The imaging performance, safety, and pharmacokinetic profile of Tsp1a-Pra-IR800 were further evaluated in non-human primates. Immunochemical studies of the peripheral nerves of grivets (*Chlorocebus aethiops*) confirmed the localisation of Na_V_1.7 in the PNS, including dorsal root ganglions, recurrent laryngeal nerves, and vagus nerves. A neck dissection mimicking human thyroidectomy was performed in these primates to simulate a real surgical scenario for intraoperative nerve imaging. A single bolus IV administration of Tsp1a-Pra-IR800 was well-tolerated with a serum half-life of ~15 min. No change in behaviour or activity (oxygen saturation, heart rate, and body temperature) was observed during thyroid surgery. Tsp1a-Pra-IR800 produced the strongest fluorescent signals in the carotid arteries and jugular veins, followed by the recurrent laryngeal nerves and vagus nerves [149]. The excellent contrast between nerves and adjacent tissues lasted for at least 30 min.

In addition to its role in nociception, Na_V_1.7 is expressed in olfactory sensory neurons where it plays a key role in olfaction [150]. Loss-of-function mutations in Na_V_1.7 have been found in patients with olfactory dysfunction, including anosmia and hyposmia [136,151]. The expression of Nav1.7 in olfactory tissues presents an opportunity to develop a Na_V_1.7-targeted imaging tool for the diagnosis of smell disorders, which, in turn, detects the tissue damage caused by viral infections or inflammation. Immunohistochemical studies in mice revealed an enrichment of Na_V_1.7 in the olfactory nerve bundles located in the lamina propria [152]. Fluorescent imaging after IV injection of Tsp1a-Pra-IR800 showed that the majority of the fluorescence accumulated in the olfactory bulb and epithelium of wild-type mice but not olfactory-ablated mice [51]. Overall, Tsp1a-Pra-IR800 could be used as a non-invasive diagnostic tool to assess the extent of damage in the nasal cavity.

In summary, Tsp1a-Pra-IR800 demonstrates remarkable nerve specificity, exceptional contrast, and an absence of side effects in animal models. Clinical translation of this probe might help to minimise nerve damage during surgery.

### 3.4. Development of Nerve-Targeted Imaging Agents

Several recent proof-of-concept studies reported the use of Na_V_1.7-targeted spider-venom peptides to highlight the peripheral nerves in animal models. From a clinical perspective, two major concerns that need to be considered for Na_V_1.7-targeted venom peptides (and by extension, other nerve-targeted agents) are the route of administration and dosage. In general, nerve-imaging agents can be administered locally or systemically [153]. Local administration involves the direct injection of the agent either into the distal target of nerves, allowing it to travel in a retrograde manner and label the nerves, or into the target nerve fibres in retrograde and anterograde directions. This approach minimises the overall dose and the risk of systemic toxicity. Nonetheless, the target nerve needs to be well-exposed, and adjacent tissues may be unintentionally labelled by the fluorophore. In contrast, systemic administration provides wide coverage of nerve fibres throughout the body. IV injection of nerve-imaging agents prior to surgery allows more time to develop contrast in the target tissue and wash out nonspecific fluorescence. The relative ease of IV injection in a preoperative setting makes it attractive for the delivery of imaging agents. Given that the whole body is exposed to the injected probe, it is crucial to evaluate the long-term retention and safety (e.g., biodistribution, pharmacokinetic clearance, and metabolism) before clinical translation.

Determining the optimal dose depends on several factors, including the optical properties of the probe, the route of administration, the imaging instrument, and the type of surgery. In contrast to the therapeutic utility of Na_V_1.7 inhibitors, which are typically dosed repeatedly over extended periods of time, a single dose of imaging agent is sufficient at the time of surgery. It is important that Na_V_1.7-targeted imaging agents do not cause acute or chronic pharmacodynamic effects. This is achieved by administering a lower dose of imaging agent that produces sufficient fluorescent contrast relative to the background and lasts for the duration of the surgery while simultaneously not inhibiting large portions of the channel to maintain its normal neuronal function in patients. The use of brighter fluorophores offers the advantages of performing fluorescent imaging at lower doses and reducing the duration of exposure.

One of the challenges for developing fluorescently labelled probes is that the addition of the fluorophore can alter the selectivity and affinity of the targeting component. The large hydrophobic π-conjugated systems in most fluorophores are susceptible to aggregation in aqueous solutions; this can cause nonspecific hydrophobic stickiness, which might affect its migration along nerve fibres. In addition, fluorophores with high lipophilicity might lead to nonspecific uptake and thereby decrease fluorescent contrast. Due to the inability to predict how modifications will affect the probe’s activity, researchers often face a trial-and-error process to explore various fluorophores and labelling methods in order to minimise loss of activity while achieving the desired fluorescent properties. This unpredictability significantly hampers the efficiency and progress of developing fluorescently labelled imaging agents for the clinical translation. Overcoming these barriers might lead to the validation and regulatory approval of a wider range of fluorescent probes for image-guided surgery.

## 4. Potential Applications of Molecular Imaging

Neurodegeneration is a process in which cells in the brain or spinal cord deteriorate over time, resulting in irreversible neuronal damage. There are numerous factors that cause this process, such as infections, free radical production, and apoptosis [154,155]. Neurodegeneration is common to many disorders such as Alzheimer’s disease (AD), multiple sclerosis (MS), and Parkinson’s disease. The neurodegenerative process occurs in different brain regions with varying degrees of clinical manifestations, most of which have catastrophic consequences for affected individuals. In the case of AD, there is an accumulation of abnormal proteins that aggregate within the brain’s limbic, paralimbic, and neocortical regions [156]. These areas are responsible for memory function. As a result, individuals experience a gradual deterioration of memory and cognitive abilities, disorientation concerning time and space, and impaired decision-making. MS is an autoimmune disease characterised by immune-mediated demyelination and axonal damage in the brain and spinal cord, leading to progressive motor, sensory, and cognitive disabilities [157]. As the disease progresses, individuals become paralysed and require assistance with daily activities.

A wide range of PET-based molecular imaging agents are currently available for the diagnosis and assessment of treatment efficacy for AD [158]. Meanwhile, various molecular imaging agents have been developed for preclinical research to investigate the underlying molecular deficit related to neurodegeneration, particularly neuroinflammation, which plays a key role in the initial onset of degenerative conditions. One promising target is the peripheral benzodiazepine receptor (PBR), which is found at high levels in activated microglial cells associated with neuroinflammation [159]. [^11^C]PK11195 is a selective PET radioligand for PBR, and [^11^C]PK11195 labelling correlates with microglial activation in transgenic mice with AD [160]. Thus, PBR represents an in vivo marker for neuroinflammatory and neurodegenerative conditions, and [^11^C]PK11195 has been used to visualise increased PBR expression in patients with MS and AD [161,162].

Acid-sensing ion channel 1a (ASIC1a) is a proton-gated sodium channel that contributes to various physiological process in the central nervous system, including neuronal excitability and synaptic transmission. The primary function of ASIC1a is to sense and respond to changes in extracellular pH. ASIC1a is activated by acidic conditions, and thus the tissue acidosis that occurs during pathological processes such as tissue damage, inflammation, and ischemia can activate ASIC1a [163]. ASIC1a is present in heart muscle cells (cardiomyocytes) where it plays a central role in ischemic injuries of the heart such as that which occurs during myocardial infarction (MI) [164]. The most potent known inhibitor of this ion channel is peptide Hi1a from the venom of an Australian funnel-web spider [165], and it was recently shown that following MI, fluorescently labelled Hi1a (Hi1a-AF700) binds in an ASIC1a-dependent manner to the injured myocardium rather than healthy tissue [166]. This suggests that Hi1a could be used as tracer to monitor infarct development during MI and possibly ischemic injuries of other organs.

Traditional methods for detecting and studying MS have involved lumbar puncture and a range of imaging techniques such as MRI and PET. However, these approaches do not provide information about anatomical and physiological changes at the molecular level. Fluorescently-labelled venom peptides such as Hi1a that specifically bind ASIC1a might allow visualisation of the expression levels and distribution of ASIC1a in MS patients, which might provide insights into the underlying mechanisms of MS. In this way, researchers could track the activation and involvement of ASIC1a in neuroinflammatory processes in the brain. This could help elucidate the specific molecular pathways through which ASIC1a modulates inflammation and identify potential targets for therapeutic intervention. However, the poor BBB penetrability of most venom peptides presents a major challenge for imaging receptors and ion channels in the CNS.

## 5. Conclusions

During hundreds of millions of years of evolution, natural selection has favoured the accumulation of venom toxins that modulate the function of membrane receptors and ion channels with high potency and selectivity. Venom-derived peptides have long been used as imaging tools for elucidating the role of ion channels within complex biological systems. Recent advances in optical imaging techniques and the increasing availability of clinically approved fluorophores have expanded our ability to visualise targets of interest. Although venom-derived peptides have shown promise in preclinical studies, they have not yet achieved the same level of validation and acceptance as existing imaging agents. Despite their numerous advantages, further research and development are needed to optimise the design and clinical translation of venom-derived peptides for intraoperative imaging. Nevertheless, the typical challenges associated with peptide-drug development, such as large-scale manufacture and investigational new drug-enabling preclinical toxicology studies, seem readily surmountable for venom-derived imaging agents because they are used once-off in small amounts for in vivo imaging applications. A larger challenge will be their application to CNS targets, but the success of CTX shows that even this barrier is not insurmountable.

## Figures and Tables

**Table 1:** Fluorescently labelled venom peptides that target ion channels.

Animal Source	Peptide	Molecular Target	Fluorophore	Reference
Cone snail	α-conotoxin TxID	α3β4 nAChR	5-TAMRA	[27]
	α-conotoxin ArIB	α7 nAChR	Cy3, Alexa Fluor 546	[28,29]
	α-conotoxin RgIA	α9α10 nAChR	Cy5	[30]
	ω-CgTx	Ca_V_2.2	TexasRed, fluorescein, Cy3	[31,32,33]
Snake	α-cobratoxin	α7 and α9/α10 nAChR	Enhanced GFP	[34]
		α1β3γ2 GABA_A_ receptor	Alexa Fluor 546	[35]
	α-bungarotoxin	α7 nAChR	Cy5	[36]
	Mambalgin-2	ASIC1a	CF647	[37]
Scorpion	ShK	K_V_1.3	F6CA	[38]
	HgTx1	K_V_ family channels	Cy3, Cy5, Alexa Fluor family	[26]
	IbTx	K_Ca_1.1	Alexa Fluor 488	[39]
	OSK1	K_V_1.1–1.3	Enhanced GFP	[40]
	AgTx2	K_V_1.1–1.3, 1.6	RFP	[40]
	CTX	Cl^–^ channels	Cy5.5, IR800, ICG	[41,42,43]
	TsTx	Na_V_ channels	Alexa Fluor 488	[44]
	TiTx-γ	Na_V_ channels	Alexa Fluor 568	[44]
	LqqV	Na_V_ channels	DACA	[45]
Spider	GsTx	K_V_2.1	DyLight 550	[46]
	DkTx	TRPV1	Fluorescein	[47]
	ProTx-II	Na_V_1.2, 1.5, 1.7, 1.8	ATTO488	[48]
	Hs1a	Na_V_1.1–1.3, 1.6, 1.7	Cy7.5, bacteriochlorin	[49,50]
	Tsp1a	Na_V_1.7	BODIPY, chlorin, IR800	[49,51,52,53]

ASIC1a, acid-sensing ion channel 1a; BODIPY, boron-dipyrromethene; Cl^–^, chloride ion channel; Cy, cyanine; DACA, 7-dimethylaminocoumarin-4-acetate; F6CA, fluorescein-6-carboxyl; GABA_A_, γ-aminobutyric acid type A; GFP, green fluorescent protein; ICG, indocyanine green; K_V_, voltage-gated potassium channel; Na_V_, voltage-gated sodium channel; nAChR, nicotinic acetylcholine receptor; RFP, red fluorescent protein; TAMRA, 5-carboxytetramethylrhodamine; TRPV1, transient receptor potential vanilloid 1.
